# Acceptability of physician associate interns in primary care: results from a service evaluation

**DOI:** 10.1186/s12875-021-01372-5

**Published:** 2021-12-20

**Authors:** Elizabeth Cottrell, Victoria Silverwood, Alex Strivens-Joyce, Lucy Minshull, John J. Edwards, Sarah Lawton, Matt Aiello, Sharon Turner

**Affiliations:** 1 Keele University, Newcastle, UK; 2 Wolstanton Medical Centre, Newcastle-under-Lyme, UK; 3 Wolstanton Medical Centre, Palmerston Street, Wolstanton, Newcastle-under-Lyme, Staffordshire ST5 8BN UK; 4 Honeywall Medical Practice, Stoke-on-Trent, UK; 5 Lead PA Montgomery Medical Practice, Montgomery, UK; 6 North Staffordshire GP Federation, Stoke-on-Trent, UK; 7 Keele Clinical Trials Unit, Keele University, Newcastle-under-Lyme, UK; 8 Urgent and Emergency Care, Health Education England, London, UK

**Keywords:** Physician associate, Internship, Primary care, Evaluation

## Abstract

**Background:**

Physician associates (PA) form part of the policy-driven response to increased primary care demand and a general practitioner (GP) recruitment and retention crisis. However, they are novel to the primary care workforce and have limitations, for example, they cannot prescribe. The novel 1 year Staffordshire PA Internship (SPAI) scheme, introduced in 2017, was established to support the integration of PAs into primary care. PA interns concurrently worked in primary and secondary care posts, with protected weekly primary care focussed education sessions. This evaluation established the acceptability of PA interns within primary care.

**Methods:**

All ten PAs from the first two SPAI cohorts, the nine host practices (supervising GPs and practice managers) and host practice patients were invited to participate in the evaluation. A conceptual framework for implementing interventions in primary care informed data collection and analysis. Data were gathered at three time points over the internship from practices, through discussions with the supervising GP and/or practice manager, and from the PAs via discussion groups. To enrich discussion data, PA and practices were sent brief surveys requesting information on PA/practice characteristics and PA primary care roles. Patient acceptability data were collected by the host practices. Participation at every stage was optional.

**Results:**

By evaluation end, eight PAs had completed the internship. Seven PAs and six practices provided data at every time point. Five practices provided patient acceptability data. Overall PA interns were acceptable to practices and patients, however ambiguity about the PA role and how best to communicate and operationalise PA roles was revealed. An expectation-preparedness gap resulted in PAs needing high levels of supervision early within the internship. SPAI facilitated closure of the expectation-preparedness gap and its funding arrangements made the high supervision requirements more acceptable to practices.

**Conclusions:**

The test-of-concept SPAI successfully integrated new PAs into primary care. However, the identified challenges risk undermining PAs roles in primary care before they have attained their full potential. Nationally, workforce leaders should develop approaches to support new PAs into primary care, including commitments to longer-term, sustainable, cohesive and appropriately funded schemes, including structured and standardised education and supervision.

## Background

Physician Associates (PAs) are dependent healthcare professionals, who have been trained in the medical model [1]. Having completed a relevant undergraduate degree, PAs must successfully complete a dedicated two-year postgraduate PA training course. Until 2018 there were fewer than 200 PAs working in UK primary care [2, 3]. PAs can work in primary care immediately upon graduation, undertaking roles such as taking histories and assessing patients, formulating management plans and managing incoming results and correspondence [4]. Roles within primary care can increase as the PA gains experience, however an accountable General Practitioner (GP) must be constantly available for clinical support and prescription authorisation [4]. There has been a dramatic policy-driven [5] increase in student PAs, from 80 students across three UK higher education institutions (HEI) in 2014 to 1800 students across 35 UK HEI in September 2019. While this positioned the workforce to deliver the NHS Long Term Plan [6] through Primary Care Networks (PCNs) [7, 8], at the time, accompanying literature detailing primary care roles of PAs was limited compared to that for secondary care [9]. Although information provision has since improved [4, 10, 11], available data remain predominantly based on USA-trained primary care PAs; a more established workforce able to prescribe and order radiographs [12], unlike UK PAs. Whilst PAs have been introduced as a rapid and relatively inexpensive way to grow the UK primary care workforce, little is known about their clinical effectiveness, potential for return on investment [13], the integration of newly qualified PAs (‘new PAs’) into the UK workforce nor their acceptability to patients and primary care healthcare professionals.

Workforce leads in Staffordshire recognised the potential for primary care PAs in 2016 to support a significant GP-workforce crisis [3]; several practices were becoming unsustainable and were facing closure. However, to promote successful, safe integration of PAs into primary care roles, a structured and supportive postgraduate programme was developed; a primary care PA internship scheme for fully qualified ‘new PAs’. Within an external evaluation designed to establish how an internship could support the integration of PAs within primary care teams, the results presented in this paper report the acceptability of PA interns in primary care.

## Methods

### The Staffordshire Physician Associate Internship (SPAI)

The innovative SPAI was created and delivered by the North Staffordshire GP Federation (NSGPF) in partnership with five NHS trusts across Staffordshire, with a single lead employer (for further information see website [14]). Investment was provided jointly by Health Education England (HEE) and NHS England (NHSE) to enable PA intern posts to be subsidised and to defray costs of training and malpractice insurance. The SPAI was designed as a single-year, pilot, test-of-concept, split-post scheme in which interns spent: half the week in primary care, 2 days in secondary care, and half a day undertaking protected learning. PA interns were intended to remain in the two clinical posts concurrently for the whole year. All PAs in the first cohort (commenced October 2017) followed this approach, however two in the second cohort (commenced March 2018) had full time primary care placements. Practices were provided with published literature available at the time [9] to support the integration of the PA into the primary care workforce, however, they were free to utilise the PA in any of the described role that best matched their practice’s needs.

A syllabus for the half-day teaching on key clinical topics relevant to primary care was developed prior to commencement of the SPAI programme. At least 75% of sessions had to be attended and recorded within a continuing professional development (CPD) tracker entry in order for an intern to be eligible for a certificate of completion of the internship programme.

Before each SPAI cohort, practices were sent information and asked to express their interest. From these expressions of interest, the SPAI team selected practices based on factors such as Care Quality Commission (CQC) performance and training experience. As far as possible, PAs were matched to practices based on their preferences and travel distance from home.

### Data collection

This service evaluation relied on voluntary participation of interns and practices. Evaluation participants were PA interns and host-practice staff and patients from the first two SPAI cohorts. To gain the required breadth and depth of information, a pragmatic approach was undertaken to gather the required data. Intern (group) and in-practice discussions (GP and/or practice manager) took place at cohort start, mid-point and end. Discussion data were enriched using brief surveys sent at the time of the discussions to obtain more detail about intern/practice characteristics, PA roles and plans for the PA over time. All PA and practice data, both verbal and written, were not anonymous and were collected by one individual (EC) whose only role in the SPAI team was to evaluate the internship and feedback findings concurrently to the wider SPAI team.

Patient acceptability data was collected by practices at one time-point (May–September 2018) using a practice-delivered patient survey. Closed questions associated with a Likert-scale, adapted, with permission, from the IPSOS Mori patient survey [15] assessed patients’ satisfaction with the intern and space for free text feedback was provided. Anonymous patient responses were forwarded to the evaluator.

**Fig. 1:**
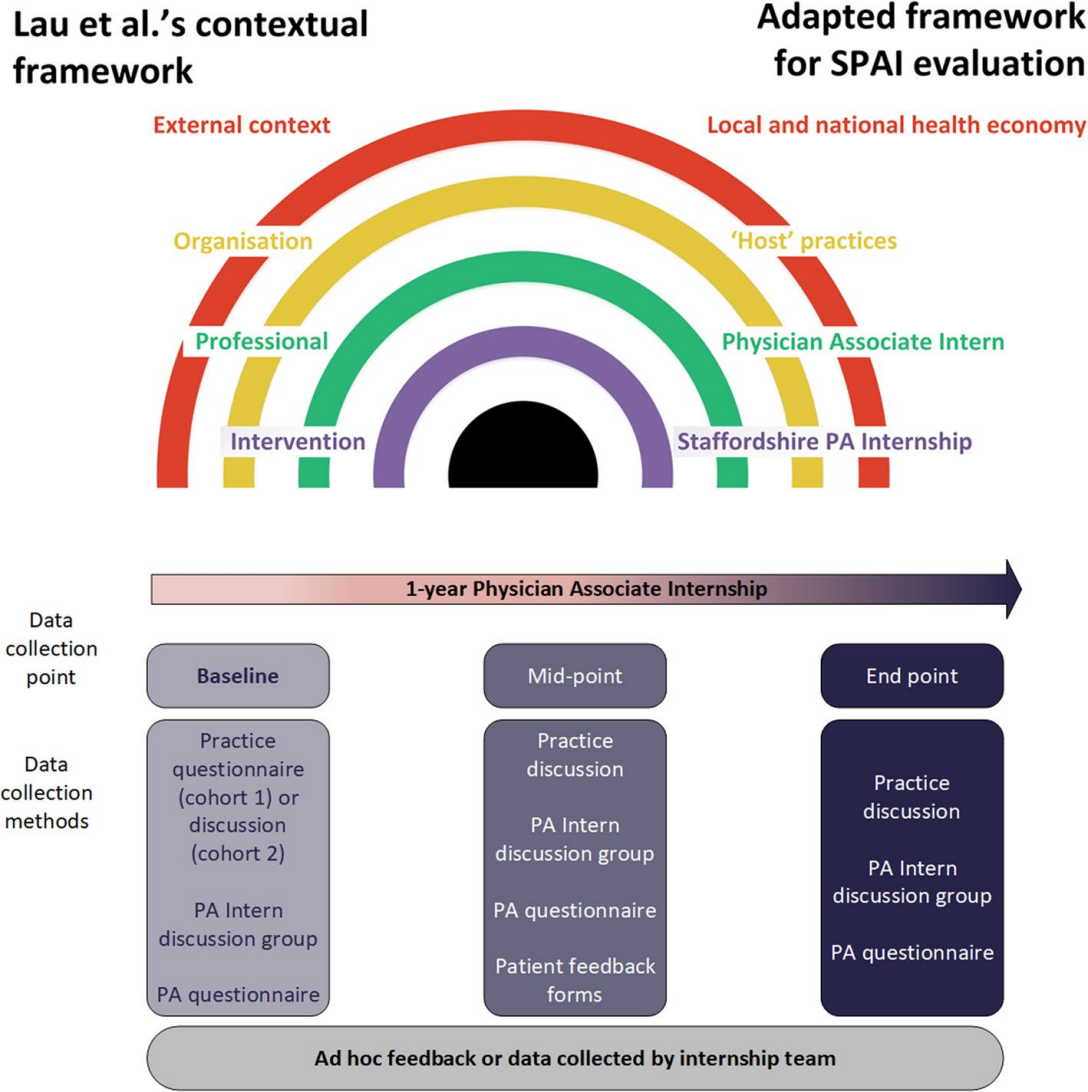
Adaptation of Lau et al’s [16] conceptual framework to the evaluation framework and overview of evaluation data collection

Evaluation data were gathered and subsequently analysed according to domains within Lau et al.’s conceptual framework for implementing interventions in primary care [16]. The conceptual framework was adapted such that ‘external context’ included local and national health economies and policy, ‘organisation’ included data relating to host-practices, ‘professional’ related to the intern and ‘intervention’ was the SPAI (Fig.1).

### Data analysis

Results from both cohorts and all sources were combined. Closed and multiple-choice survey responses were coded in IBM SPSS and descriptively analysed. Open responses that mapped to closed survey responses (e.g. staff mix) were coded and amalgamated into quantitative evaluation data-sets. Verbal data from discussion groups (interns) or meetings (individual host practices) were audio recorded and transcribed. Open responses from the discussions and intern, practice and patient survey data were combined and thematically analysed within NVIVO using our evaluation framework. Concepts drawn from these data were independently identified and coded by two evaluators (EC and VS). Our evaluation framework remained fixed, however sub-themes and concepts developed iteratively, mapped together, and areas of agreement and dissonance between respondents were identified.

## Results

Ten interns commenced across the two cohorts. Eight interns were newly qualified PAs. Among the other two, one had been working in primary care for a few months and another on a respiratory ward. One practice hosted an intern in both cohorts. At evaluation end: eight had completed the internship, one left the SPAI early for a primary care job elsewhere and one was on maternity leave. Overall, data were collected from all interns at baseline (seven gave data at every time-point), eight practices at baseline (six gave data at every time-point), and 165 patients from five practices. Key themes relating to the acceptability of interns in primary care are described below.

### Conceptualisations of newly qualified PAs

While individual PAs integrated as well-liked team members, both PAs and practices lacked clarity about what a PA is, and/or how best to communicate this to patients and colleagues. Three common conceptualisations of PA were: what they were not (e.g. not a doctor or a nurse); what they were similar to (e.g. salaried or trainee GPs, medical student); or what they could not do (e.g. they cannot prescribe). Positive conceptualisations of what they were, and positive statements about their role, were lacking.

Receptionists’ misunderstanding about interns caused inefficiencies; the ‘wrong type’ of patients being allocated to interns. Dissatisfaction arose if practices had greater expectations of autonomy (e.g. PAs are similar to a trainee GP). Although on reflection, these expectations were recognised as being unrealistic, practices highlighted existing literature outlining potential roles of (experienced) PAs [17] as contributing to these perceptions. Patients valued consultations with PAs but lacked clarity about the professional they had seen. PAs recognised this. Forty-seven (29%) responding patients reported that they did not know they were seeing a PA.

PAs considered themselves most similar to GPs, although recognised they were not GP replacements. GPs agreed that PAs adopted a similar approach to patient assessment and presentation, but thought PAs had less depth of understanding and reduced ability to diagnose and consistently manage complex conditions.

Whilst PAs had some gaps in their capabilities, compared with trained GPs (the lack of prescribing capability was mentioned repeatedly), their flexibility to perform elements of different healthcare professionals’ roles was valued by GPs and practice managers. Overall, interns were perceived as a hybrid of multiple primary care roles.

**Table:** 

*Box 1 Quotes illustrating views regarding the conceptualisations of interns*	
*“No ‘cause if we say…Physician’s Associate. And what’s one of them? [laugh]… isn’t a doctor…isn’t a nurse...in-between but he can do most things (*Sic*).”* (Practice Manager) *“I see [the intern]…Half-way between a nurse which is looking at pure clinical…whereas a GP…is…more holistic with a bit of social care added in there”* (Practice Manager) *“… [the intern is] not working as a registrar. [the intern is] working as a medical student…”* (Practice Manager) *“Like that hypothetical deductive model?...No I don’t think [the intern is] there. I think [the intern is] taught a bit like a third year medical student…these are the questions you ask about chest pain*. *.*.” (General Practitioner) *“…I really like…the flexibility of a PA. There’s nothing they can’t really do, obviously there’s the prescribing bit, but actually can they visit? Yes…Could they see children? Well yes they can in time...There’s been no resistance. [the intern has] just – well show me what to do and I’ll do it.”* (General Practitioner) *“…it said physician associate on the door and not doctor…have you not got all your stripes yet?…they keep calling you doctor and you keep saying just stop there.”* (PA intern) *“The doctor was easy to listen to and explained everything well, easy to understand, very pleased with the doctor”* (Patient)	

### Perceptions of the PA role in primary care

The lack of a clear, realistic description of the primary care roles that PAs were equipped to undertake caused uncertainty and confusion amongst some practices and patients. Practices often relied on interns to outline their own professional boundaries. Confusion over PAs being independent or dependent practitioners emerged. Practices often recognised that PAs were dependent on GPs for supervision, but lacked clarity about the wider implications of this (e.g. the process for administration of influenza vaccinations). Some practices described uncertainty among existing staff about the role(s) a PA would occupy. However, despite lack of role clarity, host-practices saw the subsidised internship as an opportunity to explore something ‘different’. Practices adapted to supervision requirements through a dynamic approach to intern tasks, generally increasing the proportion of time interns undertook patient reviews and non-clinical activities. By internship end, practices were developing conceptualisations of future, higher-level implementation of a PA.

**Table:** 

*Box 2 Quotes illustrating perceptions of the PA role in primary care*	
*“Am I utilising him correctly as the Internship is expecting us to do?”* (Practice Manager) *‘there’s sort of no singular PA job description…There’s a broad range of things that they can do but it’s making sure that the person…can do what you want them to do and they’re happy to do it.’* (Practice Manager) *“I think they bring something different… they really sit between somewhere like a nurse practitioner and a junior doctor…when you get a very experienced PA, then they certainly are going to be like a good junior doctor.”* (Practice Manager) *“…there’s lots of things that they can assist us with…things like phoning patients on our behalf…rather than just seeing their own patients – helping us with our patients…”* (General Practitioner)	

### Preparedness of interns for primary care: the expectation-preparedness gap

This evaluation uncovered a significant expectation-preparedness gap. All PAs expected to undertake book-on-day (BOD) appointments in primary care, though only 44% came into the internship with this experience. Similarly, 89% of interns had expected to do long-term condition reviews but only 22% had prior experience (Table 1). Interns were under-equipped on internship commencement to manage primary care patients with undifferentiated, complex, multi-morbidity.

**Table 1:** Expected and actual roles compared to previous experience in primary care – the expectation-preparedness gap

Potential roles	Experience prior to Internship (*n* = 9)	Expectation-preparedness gap	Expected to do at baseline (*n* = 9)	Undertaking at end of the Internship (*n* = 7)	
Clinical contact activities					
Book-on-day (acute) appointments	4 (44%)		9 (100%)	6 (86%)	↓
Long term condition reviews	2 (22%)		8 (89%)	5 (71%)	↓
Home visits	3 (33%)		7 (78%)	6 (86%)	↑
Pre-booked (routine) appointments	5 (56%)		6 (67%)	5 (71%)	↑
Visits to care/nursing homes	2 (22%)		5 (56%)	6 (86%)	↑
Minor surgery	1 (11%)		2 (22%)	0 (0%)	↓
Duty (on-call) roles	0 (0%)		0 (0%)	0 (0%)	←→
Activities outside of clinical contact					
Results handling	2 (22%)		8 (89%)	3 (43%)	↓
Processing incoming letters/reports	0 (0%)		6 (67%)	3 (43%)	↓
Generating outbound letters/reports	0 (0%)		6 (67%)	2 (29%)	↓
Quality improvement activities	0 (0%)		4 (44%)	5 (71%)	↑
MDT meetings	2 (22%)		4 (44%)	3 (43%)	←→
Teaching others	0 (0%)		1 (11%)	3 (43%)	↑

### Acceptability of the intern among the primary care team

Upon internship completion, most host-practices were overwhelmingly positive about their PA; they demonstrated good clinical skills and performed well in a variety of scenarios. Acceptability of PAs grew as initial reservations from some primary care team members eased. Reservations often arose from nursing staff and were related to potential implications of unclear role boundaries. Other concerns, from GPs, included issues regarding prescribing, accountability and supervision time pressures. Practices with previous experience of PAs (usually as students), felt that this enhanced acceptability of the intern amongst staff. PA acceptability among practices was not always driven by increased clinical contact capacity. Pressure in GPs’ working days includes the ‘silent workload’; the administrative workload undertaken around full clinics, often unseen by patients and some staff. Introduction of the intern, and resultant increased requirement for supervision and blocked appointments, provided some additional time for GPs to undertake this workload. Further, the PAs undertook some of the GPs’ silent workload.

**Table:** 

*Box 3 Quotes illustrating views regarding the acceptability of the intern among the primary care team*	
*“…I actually sat watching [the intern] do a couple of quite challenging learning disability reviews which I would never have the patience to do the way [the intern] did…[the intern] was very good and I was thinking…I’d be racing through this so the patient probably had a better deal.”* (General Practitioner) *“That’s what our nurse was asking as well. What is it [the intern] will do? And I couldn’t answer that question. I said time will answer I think”* (General Practitioner) *“I don’t think the nurses were keen….And I still don’t think they’re keen. I think they feel slightly threatened…and perhaps they feel [they] are better qualified…but, it’s another skill mix isn’t it?”* (Practice Manager) *“I mean [the GP] and myself often refer to the silent workload…GP’s have got a silent workload of the prescribing…the referrals, etc….there’s a huge silent workload for the practice…all these clinical audits and stuff that requires clinical input but not particularly a GP…that’s where [the intern has] been so useful”* (Practice Manager)	

### Acceptability of supervising the PA intern

PA intern supervision, and the related issue of trust, were important topics to practices and interns. High-intensity, close supervision was necessary for responsible GPs to build trust in the interns’ capabilities. Perceptions of acceptable supervision levels appeared to be individual GP and/or practice-specific. A single salaried GP in a host-practice refused to provide clinical supervision for the intern due to perceived risks. Some GPs reviewed all intern patients for an extended period but felt over-burdened by this. Conversely, after a short induction period (a few weeks), other GPs allowed supervision to be determined by the intern and case complexity. Whilst training practices were more likely to find the supervision demands acceptable, they often had multiple dependent practitioners/trainees concurrently requiring GP support. The PA intern was the tipping point in some practices, who adapted their working day (e.g. reducing booked appointments) to accommodate this. Notably, multiple people requiring support from one GP created delays for some interns. Smaller practices struggled most to provide ‘blocked-out’ appointments to accommodate ‘just-in-time’ supervision, due to the relative impact on appointment capacity. The consequences of this were that PAs sometimes rescheduled patients with another clinician to complete the management plan and/or felt less supported. Practices that had invested a lot of high-level supervision early on appeared more satisfied at internship end. Over the year, GP face-to-face reviews reduced, and practices and interns developed efficient and sustainable supervision and support methods which, in turn, increased acceptability of supervision. For example, protocolising care for common conditions; utilising electronic prescribing systems (sending electronic prescription requests) if no advice was needed; use of medical record screen messaging systems for brief advice; joint debrief sessions with other dependent professionals/trainees; and catch-up meetings. A mismatch in perceptions of appropriate supervision emerged: some interns felt over-observed and others under-supported.

**Table:** 

*Box 4 Quotes illustrating the acceptability of supervising PA interns*	
*“at the moment, it’s not really working for the GPs because so much of their time is going in training…At the moment it’s more work. ..”* (Practice Manager) *“…what I think would be more effective is if all the GP practices had one on-call doctor that wasn’t seeing any patients so PAs could go to that doctor ‘cause I think waiting round outside doors, knocking the doors, waiting for patients to leave – it takes consultation time and it also saves us waiting around and feeling awkward…”* (PA intern) *“From my perspective it’s definitely been manageable. I think there’s a strong argument that [the intern] should get more input than [they get] really.”* (General Practitioner)	

### Fit of the interns with existing primary care services

The fit within the primary care team was challenged by imprecision in the PA interns’ roles and conceptualisations. However, intern role-flexibility overcame this. PAs (and practice staff) recognised that their activities were a hybrid of GP and nursing roles and the interns’ flexibility was highly valued and nurtured in many practices. However, in one practice it prevented identification of a specific role for the PA post-internship: *“So I can’t say we’ve had a bad experience but I can’t say yes, we really want one, we can’t do without one. I don’t think we’ve found that role to fit [the intern] in.”*

The inability of interns to prescribe was a commonly cited barrier to integration and certainty of the relative benefit of PAs compared with other professional groups (e.g. Nurse Practitioners). One practice felt that regular contact with GPs through prescription signing, slowed the intern’s progression towards autonomy.

While the SPAI team provided guidance about target appointment lengths, in reality this was predominantly led by the interns’ preferences. Interns were over-optimistic at the outset about the rate at which their appointments would reduce in length (Table 2). Persistence of longer appointment times was attributed to lack of patient and intern knowledge about PA appointment time norms, the need for interns to seek GP input for prescriptions, the undifferentiated nature of primary care patients, being managed in non-protocol-driven ways, and the need for longer appointments for certain activities/reviews. Despite this, GPs perceived a pressure to reduce appointment lengths to demonstrate acceptable value for money beyond the internship.

**Table 2:** PA intern reported appointment length at each time point alongside their baseline predictions for appointment length at 3 month

	PA reported appointment length at baseline (*n* = 10)	PA baseline prediction of appointment length at 3 months (*n* = 10)	PA reported appointment length at midpoint (*n* = 7)	PA reported appointment length at endpoint (*n* = 7)
30 min	8	2	0	1
20 min	2	2	7	4
15 min	0	6	0	2
10 min	0	0	0	0

Some interns enhanced care by improving outreach to housebound patients, for example, when host-practice nurses did not do home visits, interns did long-term condition reviews at home, and proactive reviews and clerking of nursing/care home admissions. These host-practices noted better or easier attainment of incentivised targets.

### Acceptability of interns to patients

Most patients had confidence and trust in the PAs (Table 3); the free-text comments provided by patients indicated that positivity stemmed from PA interns:

- Having protracted appointment length and thus they did not feel rushed and had had a thorough assessment
- Demonstrating a caring and listening approach
- Seeking second opinions when needed

**Table 3:** Patient feedback regarding the care they experience from PA interns

Aspect of care (no. of respondents)	(Very) good	Neither good nor poor	(Very) poor
Overall, how would you describe your experience with the PA? (*n* = 160)	158 (99%)	1 (< 1%)	1 (< 1%)
How good was the PA at…			
…giving you enough time? (*n* = 160)	160 (100%)	0	0
…treating you with care and concern? (*n* = 160)	158 (99%)	1 (< 1%)	1 (< 1%)
…listening to you? (*n* = 160)	157 (98%)	3 (2%)	0
…explaining tests and treatments? (*n* = 158)	155 (98%)	2 (1%)	1 (< 1%)
…involving you in decisions about your care? (*n* = 159)	154 (97%)	4 (3%)	1 (< 1%)

Patient acceptability was also indirectly indicated through repeat appointments with interns. One patient was dissatisfied by being care-navigated to the PA, but this related to the patient’s preference for a GP rather than the care provided by the PA per se. Some practices noted the value of proactive intern promotion of patient acceptance: engaging the patient participation group (PPG) and developing a leaflet. PPG members needed reassurance about the origins of the internship (i.e. workforce development), rather than a practice-level cost-cutting exercise. Some large teaching host-practices believed that patients generally accepted that they may see a variety of professionals.

### Solutions offered to improve acceptability of interns

Host-practices saw PAs as an acceptable addition to the primary care workforce. They stressed that, to optimise the primary care PA value and acceptability, a continued commitment from NHSE and HEE and accurate, realistic information for practices was needed. This should include recognition of, and support for, high-intensity GP supervision for new PAs. A commitment to a national scheme was requested, to provide standardised education, practical and financial support to make the integration of PAs into primary care a success. This was felt to be necessary for at least a few years, until a critical mass of experienced primary care PAs is realised. Host-practices wanted a collaborative network to develop a shared understanding and standardised approaches to supporting their interns.

**Table:** 

*Box 5 Quotes illustrating solutions offered by practices to improve acceptability of PAs interns*	
*“NHS England need to stop looking short-term. If they’re gonna make a PA a proper role, and why wouldn’t they, then they need to build that into their kind of workforce modelling and look at five or 10 years not 18 month rolling.”* (General Practitioner)	

## Discussion

PAs are part of the UK primary care workforce. Interns were acceptable and had a positive impact within the primary healthcare team, particularly due to their flexibility towards their roles and activities, however, they were not ready for quasi-autonomous primary care work. High-intensity supervision in the early months increased short-term burden on practices. Further, interns’ dependent status carried risks and responsibilities for supervising GPs. These could prove unacceptable to practices with limited or overstretched GP capacity. Smaller and non-training practices reported the most difficulty with providing high-intensity supervision.

No clear definition or description of a PA was provided, in particular their specific role and professional ‘identity’. This was compounded for interns who, from the host-practice perspective, were working in an unfamiliar role (intern) and in a novel support model (the SPAI). Patient feedback indicated that, whilst patients were accepting of the professional they consulted, they lacked understanding about PAs. This opacity proved confusing for the wider practice team during care navigation. The need for a change in traditional, GP-focussed patient attitudes and workforce culture was highlighted. Disseminating the unique strengths of the PA role may help to address this.

There is potential dissonance between the apparent drivers of patient satisfaction (e.g. prolonged appointments, provision of second opinions) and drivers for practices wanting an intern (to increase capacity). It remains unknown whether such high patient acceptability will persist outside of the SPAI for new PAs and optimisation of cost-per-contact by reducing appointment times.

### Comparison with existing literature

This evaluation echoes previous descriptors of primary care PAs supporting, rather than replacing, GPs and as being acceptable to patients and professionals [18, 19]. However, this evaluation presents a slightly different view of their primary care activities, previously documented as predominantly acute assessment roles [18]. Within the SPAI, practices had shifted intern activities away from solely BOD appointments to reduce supervision burden. While existing literature identifies PAs as providing more care for less outlay, compared to GPs, costs associated with GP interruptions, the necessary supervision, and ongoing need for GPs to sign prescriptions importantly were not accounted for [18]. This evaluation suggests these factors are likely to impact on economic benefits of new PAs within primary care. The provision of adequate ‘just-in-time’ supervision is beneficial in the primary care setting [20] and should be recognised for such developmental roles. Although existing literature outlines the benefit of the flexibility of roles that primary care PAs are capable of undertaking [18], there is limited information elsewhere reporting primary care roles outside of acute assessment. This is notable given the broad range of clinical and non-clinical activities undertaken by interns in this evaluation, and so should be factored into future economic analyses and descriptors of the role of primary care PAs. The identified expectation-preparedness gap was not previously highlighted and was possibly magnified by national primary-care facing documentation [17] describing the role of established primary care PAs, possibly in contexts in which PAs are afforded wider rights. Further, confusion between dependent and quasi-autonomous working of PAs may also have been perpetuated by national descriptions of the role [21].

### Strengths and limitations of this study

This service evaluation is valuable as it describes the novel SPAI for new PAs entering primary care and ongoing feedback to relevant stakeholders has enabled findings to shape future internship cohorts and new internships in real time. This evaluation builds on previous empirical work to develop an understanding of the UK context which is crucial at this time of significant policy-driven change. All interns provided data at least once, and only one practice did not participate. Limitations are that participants were not required to complete every element of data collection, that patient satisfaction data were obtained by only five of the eight practices, and that the pragmatic design (adopted to maximise engagement) meant that exact resource use (both time and financial) could not be evaluated.

### Recommendations for practice and policy

With appropriate supervision and support to develop as healthcare professionals, new PAs appear to be a suitable and acceptable addition to the UK primary care workforce. As of 2020 the General Medical Council is working towards the statutory regulation of PAs. The lessons learned within this evaluation are likely to be transferrable to new PAs taking on PCN roles. To optimise the acceptability and sustainability of integrating new PAs into the national primary care workforce, central support and guidance needs further clarification including more explicit delineation of the roles of new PAs entering primary care from aspirational roles of more experienced PAs and patient facing resources for practices to disseminate to improve understanding of this newer professional role. Practices valued the outreach and sharing of learning that arose from the evaluation process itself, therefore cross programme meetings with practice staff, interns and programme team members may help to integrate this somewhat unfamiliar professional group. The PA interns required high-intensity supervision; placing new PAs into practices facing a critical workforce crisis is likely to be detrimental to both intern and practice [18]. In general, larger, training practices appeared to be the optimal host organisations for new PAs as they recognised that they had existing transferrable development skills and greater capacity to absorb high-intensity supervision. SPAI substantially mitigated against issues relating to supervision and the need to support PAs to address the expectation-preparedness through the additional resource, subsidised internship posts, and training provision and models, similar to this, may be required while this new workforce embeds into primary care nationally to support successful integration and satisfaction with this role.

## Conclusions

PA Interns working within a supported and subsidised primary care-focussed internship model were acceptable to patients and practices. However, as new PAs, they were unprepared for this role. This test-of-concept SPAI supported successful integration of new PAs into primary care. This may suggest that to support PAs to reach their full potential in primary care, commitments to longer-term, sustainable, cohesive and appropriately funded schemes, including structured and standardised education and supervision, may need to be delivered. Given the supervision required by new PAs in the internship, and the lack of clarity about their roles, without such investment, there is a risk that acceptability of PAs in primary care, and PAs’ views of primary care careers, will be undermined before the profession has attained its full potential.

## Data Availability

The datasets used and/or analysed during the current study are available from the corresponding author on reasonable request.

## Abbreviations

BOD: Book on day
GP: General practitioner
HEE: Health Education England
HEI: Higher Education Institution
NHSE(&I): NHS England (and improvement) [was NHSE at the time of the evaluation)
PA: Physician associate
PCN: Primary care network
PPG: Patient participation group
SPAI: Staffordshire Physician Associate Internship

## References

[1] Faculty of Physician Associates Royal College of Physicians (2020). Who are physician associates.

[2] Ritsema T (2018). Facult of physician associate census results 2018.

[3] NHS Digital (2020). General practice workforce - 31 March 2020.

[4] Curran A, Parle J (2018). Physician associates in general practice: what is their role?. Br J Gen Pract R Coll Gen Practitioners.

[5] NHS England. General practice forward view, vol. 2016. NHS England; 2016. p. 60. https://www.england.nhs.uk/wp-content/uploads/2016/04/gpfv.pdf

[6] NHS (2019). NHS long term plan.

[7] British Medical Association (2019). Creating and running primary care networkds (PCNs).

[8] NHS. Primary care networks. 2019, vol. 2019. Available from: https://www.england.nhs.uk/primary-care/primary-care-networks/

[9] Faculty of Physician Associates (2017). An employers guide to physician associates ( PA ).

[10] NHS England. NHS England » the physician associate will see you now – new role to assist patients in primary care. NHS England website. 2019 [cited 2020 Nov 10]. Available from: https://www.england.nhs.uk/gp/case-studies/the-physician-associate-will-see-you-now-new-role-to-assist-patients-in-primary-care/.

[11] BMA (2019). Employing physician associates in general practice.

[12] Scope of Practice Policy (2020). Physician assistants overview.

[13] Roland M. The future of primary care creating teams for tomorrow report by the primary care workforce commission. London: Health Education England; 2015. http://hee.nhs.uk/wp-content/blogs.dir/321/files/2015/02/The-future-of-primary-care-v7-webFINAL.pdf: Available from: https://www.hee.nhs.uk/sites/default/files/documents/The Future of Primary Care report.pdf

[14] North Staffordshire GP Federation. Staffordshire Physician Associate Internship. [cited 2020 Nov 10]. Available from: https://www.nsgpfed.org.uk/staffordshire-physician-associate-internship/

[15] NHS (2020). GP patient survey.

[16] Lau R, Stevenson F, Ong BN, Dziedzic K, Treweek S, Eldridge S (2016). Achieving change in primary care--causes of the evidence to practice gap: systematic reviews of reviews. Implement Sci.

[17] Royal College of General Practitioners (2017). Royal College of general practitioners position paper on physician associates working in general practice.

[18] Drennan VM, Halter M, Brearley S, Carneiro W, Gabe J, Gage H (2016). Corrigendum: investigating the contribution of physician assistants to primary care in England: a mixed-methods study. Heal Serv Deliv Res.

[19] Williams LE, Ritsema TS (2014). Satisfaction of doctors with the role of physician associates. Clin Med.

[20] Mertens F, de Groot E, Meijer L, Wens J, Gemma Cherry M, Deveugele M (2018). Workplace learning through collaboration in primary healthcare: a BEME realist review of what works, for whom and in what circumstances: BEME guide no. 46. Med Teach.

[21] Arshad N (2015). Physician associates. Br J Gen Pract.

